# Immunogenicity and Reactogenicity of SARS-CoV-2 Vaccines in Patients With Cancer: The CANVAX Cohort Study

**DOI:** 10.1200/JCO.21.01891

**Published:** 2021-11-09

**Authors:** Vivek Naranbhai, Claire A. Pernat, Alexander Gavralidis, Kerri J. St Denis, Evan C. Lam, Laura M. Spring, Steven J. Isakoff, Jocelyn R. Farmer, Leyre Zubiri, Gabriela S. Hobbs, Joan How, Andrew M. Brunner, Amir T. Fathi, Jennifer L. Peterson, Mustafa Sakhi, Grace Hambelton, Elyssa N. Denault, Lindsey J. Mortensen, Lailoo A. Perriello, Marissa N. Bruno, Brittany Y. Bertaux, Aleigha R. Lawless, Monica A. Jackson, Elizabeth Niehoff, Caroline Barabell, Christian N. Nambu, Erika Nakajima, Trenton Reinicke, Cynthia Bowes, Cristhian J. Berrios-Mairena, Onosereme Ofoman, Grace E. Kirkpatrick, Julia C. Thierauf, Kerry Reynolds, Henning Willers, Wilfredo-Garcia Beltran, Anand S. Dighe, Rebecca Saff, Kimberly Blumenthal, Ryan J. Sullivan, Yi-Bin Chen, Arthur Kim, Aditya Bardia, Alejandro B. Balazs, A. John Iafrate, Justin F. Gainor

**Affiliations:** ^1^ Massachusetts General Hospital Cancer Center, Division of Hematology/Oncology, Department of Medicine, Massachusetts General Hospital, Boston, MA; ^2^ Dana-Farber Cancer Institute, Boston, MA; ^3^ Center for the AIDS Programme of Research in South Africa, Durban, South Africa; ^4^ Salem Hospital, Salem, MA; ^5^ Ragon Institute of MGH, MIT and Harvard, Cambridge, MA; ^6^ Division of Rheumatology, Allergy and Immunology, Department of Medicine, Massachusetts General Hospital, Boston, MA; ^7^ Division of Hematology, Brigham and Women's Hospital, Boston, MA; ^8^ Department of Radiation Oncology, Massachusetts General Hospital, Boston, MA; ^9^ Department of Pathology, Massachusetts General Hospital, Boston, MA; ^10^ Division of Infectious Diseases, Department of Medicine, Massachusetts General Hospital, Boston, MA

## Abstract

**PURPOSE:**

The immunogenicity and reactogenicity of SARS-CoV-2 vaccines in patients with cancer are poorly understood.

**METHODS:**

We performed a prospective cohort study of adults with solid-organ or hematologic cancers to evaluate anti–SARS-CoV-2 immunoglobulin A/M/G spike antibodies, neutralization, and reactogenicity ≥ 7 days following two doses of mRNA-1273, BNT162b2, or one dose of Ad26.COV2.S. We analyzed responses by multivariate regression and included data from 1,638 healthy controls, previously reported, for comparison.

**RESULTS:**

Between April and July 2021, we enrolled 1,001 patients; 762 were eligible for analysis (656 had neutralization measured). mRNA-1273 was the most immunogenic (log_10_ geometric mean concentration [GMC] 2.9, log_10_ geometric mean neutralization titer [GMT] 2.3), followed by BNT162b2 (GMC 2.4; GMT 1.9) and Ad26.COV2.S (GMC 1.5; GMT 1.4; *P* < .001). The proportion of low neutralization (< 20% of convalescent titers) among Ad26.COV2.S recipients was 69.9%. Prior COVID-19 infection (in 7.1% of the cohort) was associated with higher responses (*P* < .001). Antibody titers and neutralization were quantitatively lower in patients with cancer than in comparable healthy controls, regardless of vaccine type (*P* < .001). Receipt of chemotherapy in the prior year or current steroids were associated with lower antibody levels and immune checkpoint blockade with higher neutralization. Systemic reactogenicity varied by vaccine and correlated with immune responses (*P* = .002 for concentration, *P* = .016 for neutralization). In 32 patients who received an additional vaccine dose, side effects were similar to prior doses, and 30 of 32 demonstrated increased antibody titers (GMC 1.05 before additional dose, 3.17 after dose).

**CONCLUSION:**

Immune responses to SARS-CoV-2 vaccines are modestly impaired in patients with cancer. These data suggest utility of antibody testing to identify patients for whom additional vaccine doses may be effective and appropriate, although larger prospective studies are needed.

## INTRODUCTION

SARS-CoV-2 has led to almost 200 million recorded infections and nearly 5 million deaths globally as of September 2021 (WHO COVID-19 dashboard). Patients with cancer have a particularly high risk of poor outcomes from SARS-CoV-2 infection with increased rates of severe disease and death.^1,2^

**CONTEXT**

**Key Objective**
To understand the immunogenicity and reactogenicity of SARS-CoV-2 vaccines in patients with cancer.
**Knowledge Generated**
Prior SARS-CoV-2 infection, vaccine type (mRNA1273>BNT162b2>Ad26.COV2.S), receipt of chemotherapy, corticosteroids, and immune checkpoint blockade were associated with immunogenicity to SARS-CoV-2 vaccines in patients living with cancer, in whom responses were overall lower than those in healthy controls. Reactogenicity was similar to reports in healthy individuals and was more frequent in those with prior infection. Systemic reactogenicity correlated with immunogenicity. Additional vaccine doses appeared safe and immunogenic.
**Relevance**
In patients with cancer, SARS-CoV-2 vaccines appear safe and immunogenic in most patients. Antibody testing may help identify those with inadequate responses, in whom additional vaccine doses appear to be safe and effective.

In clinical trials, vaccination with mRNA-1273^3^ (Moderna, Cambridge, MA), BNT162b2^4^ (Pfizer BioNTech, New York, New York), and Ad26.COV2.S^5^ (Johnson & Johnson Janssen, Leiden, the Netherlands) was efficacious in reducing the risk of severe disease and infection. Cross-trial comparisons are limited by differences in study design, and there are limited real-world effectiveness data comparing all three vaccines. A growing body of data suggest antibody and, more so, neutralization titers correlate with protection against infection after vaccination.^6,7^

Aberrant immune responses in the setting of underlying cancer, use of immunosuppressive anticancer therapies, older age, and high rates of comorbidities may collectively lead to impaired immune responses and altered reactogenicity following immunization against SARS-CoV-2. However, published trials did not specifically include patients with a history of or active cancer, although these individuals comprise > 15% of people age older than 65 years.^8^ Some studies have suggested lower seroconversion rates and antibody concentrations following SARS-CoV-2 vaccination in patients with cancer,^9-17^ and particularly low responses in patients who have received B-cell–depleting agents. However, these studies are limited in size, thereby prohibiting key subgroup analyses, and frequently report only measurement of binding antibodies, or focus on the effects of individual vaccines.

We aimed to identify correlates of the immunogenicity and reactogenicity of current US Food and Drug Administration Emergency Use Authorization vaccines in a large cohort of approximately 1,000 patients enrolled in the Cancer, COVID, and Vaccination (CANVAX) study.

## METHODS

### Study Design, Eligibility, and Study Procedures

The CANVAX study is a prospective cohort study that enrolled adult patients at the Massachusetts General Hospital Cancer Center who intended to receive or had received SARS-CoV-2 vaccination. The study was advertised on a website and on posters across the cancer center; patients were also directly referred by their oncology care team. Written informed consent was obtained. Participants completed a standardized electronic or paper questionnaire that included questions about baseline demographics, cancer treatment history, medical history, SARS-CoV-2 exposures and infection, vaccination information, and postvaccine symptoms (vaccine reactogenicity). Additional clinical information was abstracted from the medical record, including cancer type, cancer history, complete blood counts obtained at the last visit before vaccination, cancer therapy within 1 year before enrollment, or contemporaneous corticosteroid use (excluding replacement dose or chemotherapy-associated dosing).

This analysis considers CANVAX participants with completed baseline survey and antibody testing from April 21 through July 21, 2021; or with antibody testing after an additional vaccine dose through September 20, 2021. We excluded individuals who had been sampled within 7 days of the final dose of the vaccine series or had not completed the full series. The results of antibody testing at the primary timepoint were returned to participants. This study was approved by the Mass General Brigham Human Research Committee (2021P000746).

Data from healthy controls recruited into a separate study in Chelsea or Boston, Massachusetts, and analyzed contemporaneously (detailed elsewhere^18,19^) are included as healthy (noncancer) comparison cohorts.

### Antibody Assays

Serum antibody assays were performed with the Roche Elecsys Anti–SARS-CoV-2 S assay (Roche Diagnostics, Indianapolis, IN), at the Massachusetts General Hospital Core Clinical laboratory, a CLIA laboratory. Participants with a negative test result (cutoff index < 0.4) were offered confirmatory testing 7-14 days later and referred to clinical immunology specialists for further counseling at the discretion of the treating oncologist. A positive antinucleocapsid antibody (Roche Elecsys Anti–SARS-CoV-2 total [nucleocapsid assay]) was regarded as evidence of prior infection.

### Assessment of Neutralization

Neutralization was measured with a SARS-CoV-2 pseudovirus neutralization assay that has been previously described.^18,20^ A pseudovirus neutralization titer 50 was calculated by taking the inverse of the serum concentration that achieved 50% neutralization of SARS-CoV-2 pseudotyped lentivirus particles entry into cells.

### Study End Point and Statistical Analysis

The primary end points of this study were immunoglobulin (Ig)A/G/M antispike antibody concentration and neutralization titers. Secondary end points included reactogenicity. A prespecified enrollment target of 1,000 was designated, and analyses according to vaccine, cancer type, treatment type, and age were prespecified. A copy of the study protocol is available from the authors. Analyses were performed in R (v4.05) using the *gtsummary* packages and lm() and glm(family = binomial) functions. We modeled log_10_-transformed antispike concentration or pseudovirus neutralization titer 50 as the dependent variable, and age, sex, ethnicity, days postvaccination, vaccine group, prior infection, cancer type, therapy type, and steroids as the independent variables. All *P* values reported are adjusted (ie, multivariate) except in Tables S1, S7, and S8 in the Data Supplement (online only), where they represent simple Fisher's exact or chi-squared test results. Figures were rendered in GraphPad Prism v9.0.

## RESULTS

### Patient Characteristics

Between April 29 and July 20, 2021, 1,622 patients were screened for enrollment (Data Supplement) and 1,001 were enrolled. In total, 762 were eligible for the current analysis. Pertinent demographic, cancer, and therapy characteristics of the patients included in this cohort are summarized in Table 1. Of note, 9% of the cohort was non-White and 2.2% self-identified as Hispanic or Latinx. Seventy-one-and-one-half percent (71.5%) of the cohort received care primarily for a solid-organ malignancy, 11% for a hematologic malignancy, and 15% had undergone bone-marrow transplant for a hematologic indication and were classified as a separate group. Twenty-seven percent of patients were not receiving cancer-directed systemic therapy. 37.8% (288 of 762) of the participants had completed a vaccine series with mRNA-1273 (two doses), 50.3% with BNT162b2 (two doses), and 11.9% with Ad26.COV2.S (single dose). Details for each group are included in the Data Supplement. Participants were sampled at a median of 79 days (interquartile range [IQR], 57-106 days) after the first dose of vaccine.

**TABLE 1.**
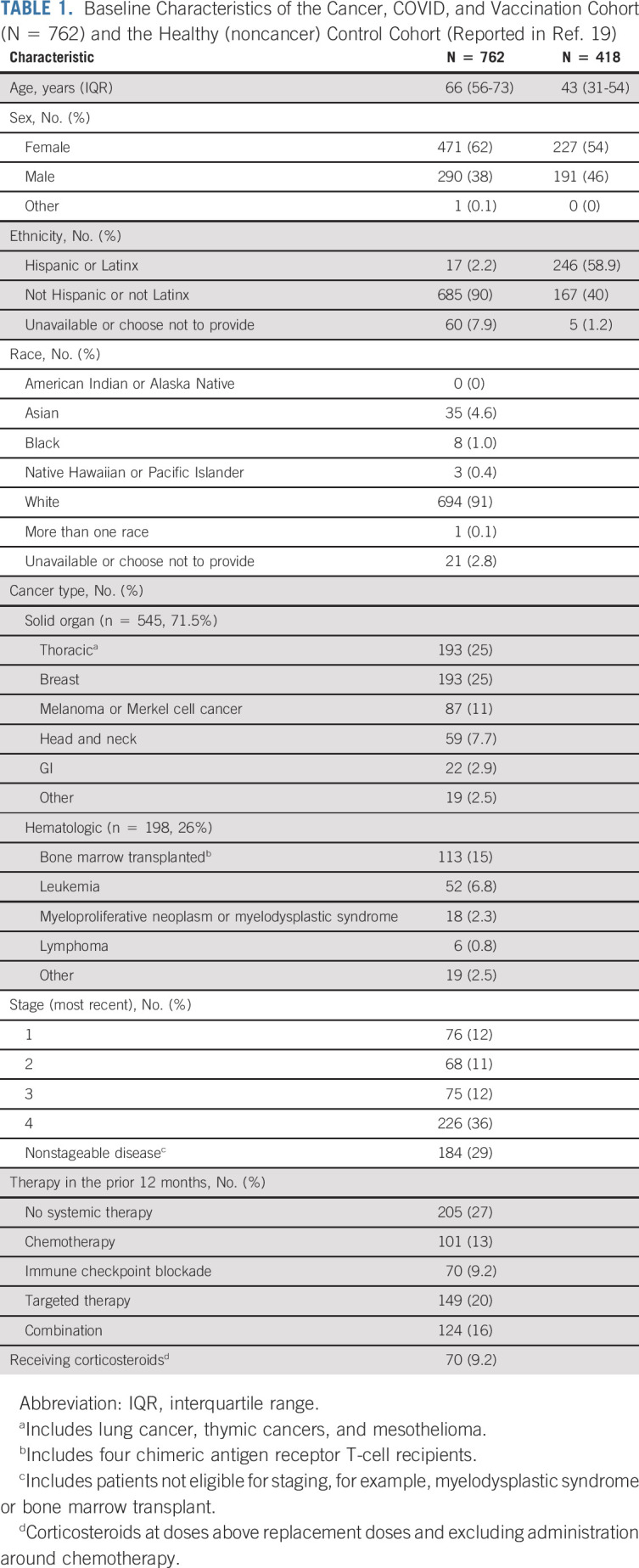
Baseline Characteristics of the Cancer, COVID, and Vaccination Cohort (N = 762) and the Healthy (noncancer) Control Cohort (Reported in Ref. 19)

### Prevalence of Antibody-Confirmed Prior SARS-CoV-2 Infection

Among the 7% (53 of 762) of participants with a positive antinucleocapsid antibody result indicating prior SARS-CoV-2 infection, 71.7% (38 of 53) reported that they had a known history of SARS-CoV-2 infection, of whom 18.4% (7 of 38) had been hospitalized and 13.2% (5 of 38) were asymptomatic. The overall proportion of asymptomatic, antibody-confirmed SARS-CoV-2 infection was 30.2% (16 of 53). Ten participants reported a prior history of SARS-CoV-2 infection but had undetectable nucleocapsid antibodies; their vaccine responses were similar to individuals without prior infection and they are analyzed as nucleocapsid antibody negative.

### Immunogenicity of SARS-CoV-2 Vaccines

Antibody responses to current US Food and Drug Administration Emergency Use Authorization SARS-CoV-2 vaccines are directed against the spike protein. We analyzed combined antispike IgA/G/M antibody concentrations and neutralization titers. For comparison, we included data using the same assays in a healthy (noncancer) cohort of 418 (supplemented further with 1,220 prepandemic controls for neutralization assay validation) healthy ambulatory adults collected contemporaneously and previously described.^19^ In the primary multivariate analysis of antibody concentration and neutralization titers, vaccine type, prior infection, treatment modalities, cancer type, age, and time of sampling are independently associated (Table 2). We present exploration of each correlate below.

**TABLE 2.**
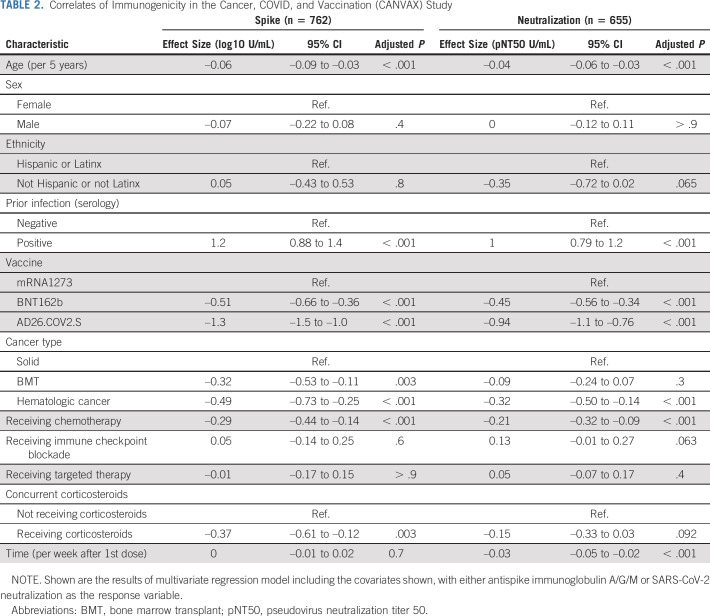
Correlates of Immunogenicity in the Cancer, COVID, and Vaccination (CANVAX) Study

#### Vaccine type and prior infection.

**FIG 1.**
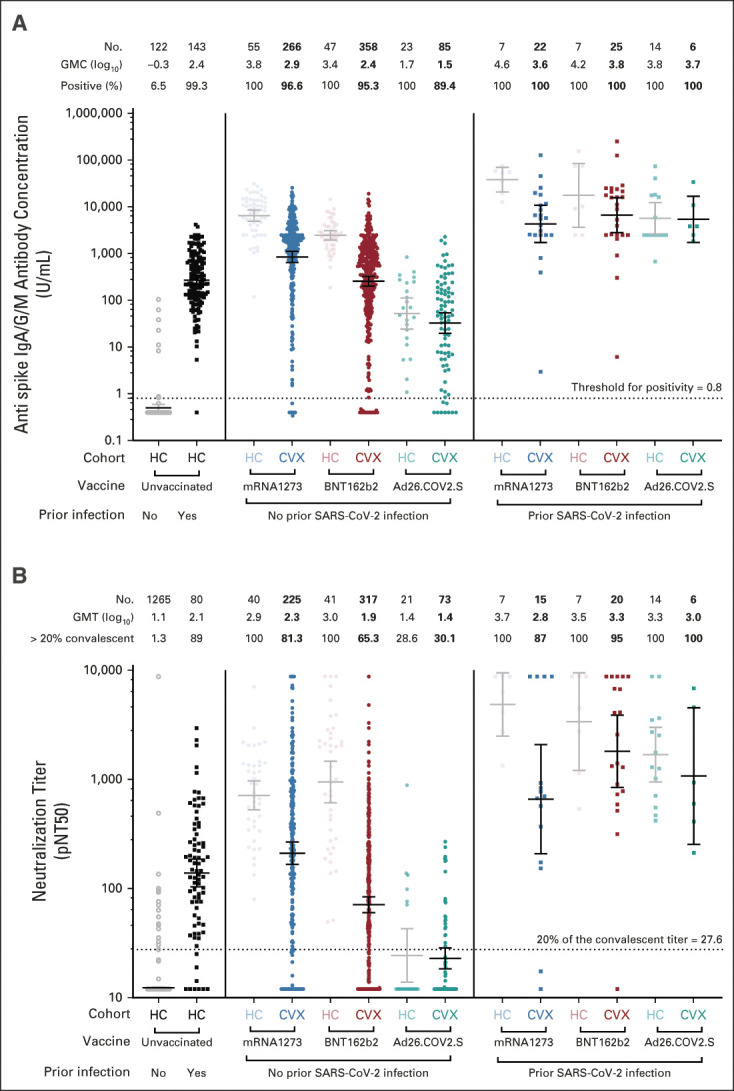
Immunogenicity of mRNA-1273, BNT-162b2, and Ad26.COV2.S in CANVAX participants. (A) The quantitative SARS-CoV-2 spike IgG/A/M antibody concentration (Roche Elecsys Anti–SARS-CoV-2 assay) in U/mL of serum for 762 CVX and 418 HCs^19^ included for interpretation. Individual measures are grouped by antibody-confirmed prior infection and vaccine. Total antispike (IgA/M/G) antibody concentrations > 2,500 U/mL triggered additional manual dilution (where sample availability allowed) to yield titers up to 250,000 U/mL. An antibody cutoff index (COI) > 0.8 was defined as positive (dotted line). All assays were run blinded to clinical information. The number of donors, GMC in log_10_ U/mL, and proportion positive are shown above each group. (B) pNT50 (defined as the titer at which the serum achieves 50% neutralization of SARS-CoV-2 wild-type pseudovirus entry into ACE2-expressing 293T cells) for 656 CVXs and 255 HCs and an additional 1,220 prepandemic controls (from Wilfredo Garcia-Beltran et al^18^) used in assay validation.^8,18^ Briefly, lentiviral particles encoding both luciferase and ZsGreen reporter genes were pseudotyped with SARS-CoV-2 spike protein (Wuhan strain) and produced in 293T cells, titered using ZsGreen expression by flow cytometry and used in an automated neutralization assay with 50-250 infectious units of pseudovirus coincubated with three-fold serial dilutions of serum for 1 hour. Neutralization was determined on 293T-ACE2 cells. A horizontal dotted line is shown at a pNT50 titer of 27.6 equivalent to 20% of the convalescent titer that is predicted to be associated with 50% protection.^6^ The number of donors, GMT, proportion with titers > 20% of the absolute geometric mean titer of convalescent healthy donors (which is 138) are shown above each group. (A and B) For each group, the horizontal line denotes the GMC or GMT, and whiskers denote the 95% CI. Corresponding statistical comparisons among CANVAX participants are as shown in Table 2; comparisons between CANVAX and healthy controls are shown in the Data Supplement. CANVAX, cancer, COVID, and vaccination; CVX, CANVAX patients; GMC, geometric mean concentration; GMT, geometric mean titer; HC, healthy control; Ig, immunoglobulin; pNT50, pseudovirus neutralization titer 50.

Antibody concentrations and neutralization titers differed significantly according to vaccine with responses to mRNA-1273 being the highest (geometric mean antibody concentration in log_10_ U/mL [GMC] 2.9; geometric mean neutralization titer in log_10_ units [GMT] 2.3), followed by BNT162b2 (GMC 2.4; GMT 1.9) and finally Ad26.COV2.S (GMC 1.5; GMT 1.4) (Fig 1, Table 2; multivariate adjusted *P* < .001). Seroconversion rates (ie, a positive spike antibody above the threshold for positivity of 0.8 U/mL) followed a similar pattern. Quantitative antibody concentrations and neutralization titers were lower in patients with cancer in CANVAX compared with healthy (noncancer) controls, even after adjusting for age, time of sampling, and vaccine (antibody concentrations: –0.6 log_10_ U/mL; 95% CI, –0.80 to –0.41; *P* < .001; neutralization titer: –0.35 log_10_ U/mL; 95% CI, –0.06 to –0.03; *P* < .0001; Data Supplement). A propensity-score matching approach yielded similar results (Data Supplement) and highlights the higher immunogenicity of mRNA vaccines compared with Ad26.COV2.S (Data Supplement). Prior SARS-CoV-2 infection was associated with significantly higher antibody titers and neutralization (Fig 1 and Table 2), as has been observed in noncancer patients.^19,21^ Relative to unvaccinated healthy (noncancer) controls with prior infection, responses among vaccinated CANVAX patients with prior infection were higher regardless of vaccine, and among those without prior infection, responses were higher after mRNA1273, similar after BNT162b2 and lower after Ad26.COV2.S (Data Supplement).

#### Therapy and cancer types.

**FIG 2.**
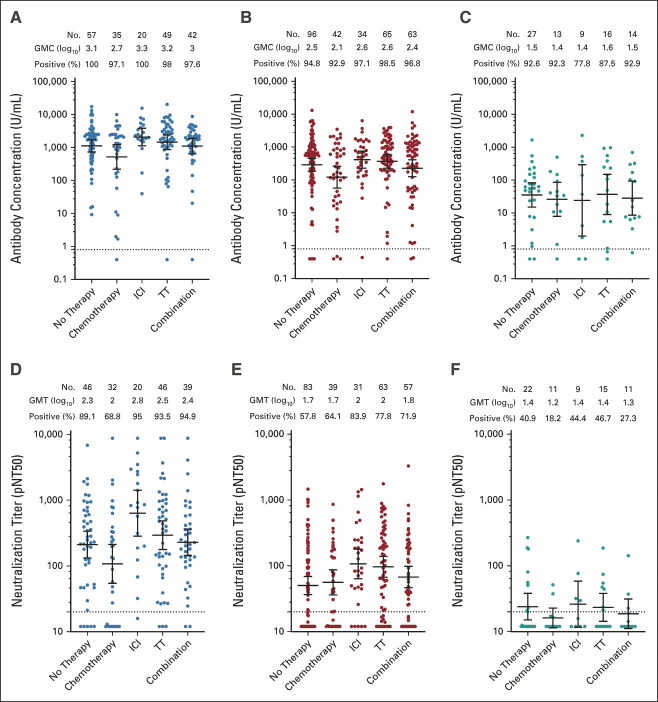
Antispike immunoglobulin A/G/M antibody concentrations (top row) and neutralization titers following mRNA-1273 (left column), BNT-162b2 (middle column), or Ad26.COV2.S (right column) according to cancer-directed therapies received in the preceding 12 months among participants without prior infection (nucelocapsid antibody-negative). Horizontal line denotes the GMC or titer and whiskers denote the 95% CI. Corresponding adjusted statistical comparisons are provided in Table 2. GMC, geometric mean concentration; GMT, geometric mean titer; ICI, immune checkpoint blockade; pNT50, pseudovirus neutralization titer 50; TT, targeted therapy.

Receipt of chemotherapy in the preceding 12 months was associated with lower antibody concentrations (–0.29 log_10_ U/mL; 95% CI, –0.44 to –0.14; *P* < .001) and neutralization titers (–0.21; 95% CI, –0.32 to –0.09; *P* < .001; Fig 2; Table 2). There was no statistical heterogeneity in effect by time of chemotherapy administration (1 month, 1-3 months, and 3-12 months; interaction *P* > .05). In a nonprespecified analysis in 458 individuals with blood counts available, lymphopenia (absolute lymphocyte count< 1,000/μL), measured at the last visit before vaccination (median of 6 days prior, IQR 19:0 days), was associated with lower antibody concentrations (–0.26 log_10_ U/mL; 95% CI, –0.46 to –0.06; *P* = .01), but not neutralization titers. Neither absolute neutrophil count nor neutropenia (absolute neutrophil count < 1,500/mL) was associated with antibody concentrations.

Receipt of immune checkpoint blockade tended to be associated with higher neutralization titers (0.13; 95% CI, –0.01 to 0.27; *P* = .063; Fig 2; Table 2). Current receipt of corticosteroids was associated with lower antibody concentrations (–0.37; 95% CI, –0.61 to –0.12; *P* = .003) and tended to be associated with lower neutralization titers (–0.15; 95% CI, –0.33 to 0.03; *P* = .09; Data Supplement). There were no significant differences in antibody concentration or neutralization titers between tumor types among patients with solid tumors, but responses were lower in patients who had undergone bone marrow transplantation (Data Supplement) or with hematologic malignancies.

#### Age and time of sampling.

Increasing age was associated with lower antibody concentration and neutralization titers (*P* < .001 for both comparisons; Data Supplement). Later sampling relative to vaccination was associated with significantly lower neutralization titers, and modeling these cross-sectional measures suggested a linear decay (Data Supplement).

#### Correlates of low neutralization.

Antibody levels and neutralization titers correlate with protection against infection in animal models^22,23^ and clinical trials of vacines.^6,7^ There is, as yet, no specific threshold indicative of protection but a neutralization titer > 20% of the GMT in convalescent individuals (a value of 27.6 in this study) corresponds with 50% reduction in infection risk in modeling studies.^6^ Overall, 18.7% of patients with cancer who received mRNA-1273, 34.7% who received BNT162b2, and 69.9% who received Ad26.COV2.S had neutralization titers lower than this level (Fig 1). Receipt of Ad26.COV2.S (odds ratio [OR] 11.3; 95% CI, 6.04 to 21.6 relative to mRNA-1273; *P* < .001), BNT162b2 (OR 2.47; 95% CI, 1.63 to 3.82 relative to mRNA-1273; *P* < .001), hematologic malignancy (OR 1.57; 95% CI, 1.05 to 2.35; *P* = .028), and age (OR 1.12 per 5-year increase; 95% CI, 1.04 to 1.21; *P* = .002) were associated with increased odds of having a low neutralization titer < 27.6 (Data Supplement). Prior SARS-CoV-2 infection (OR 0.14; 95% CI, 0.03 to 0.42; *P* = .002) and receipt of immune checkpoint blockade (OR 0.47; 95% CI, 0.27 to 0.80; *P* = .002) were associated with reduced odds.

### Reactogenicity of SARS-CoV-2 Vaccines in Patients With Cancer

**FIG 3.**
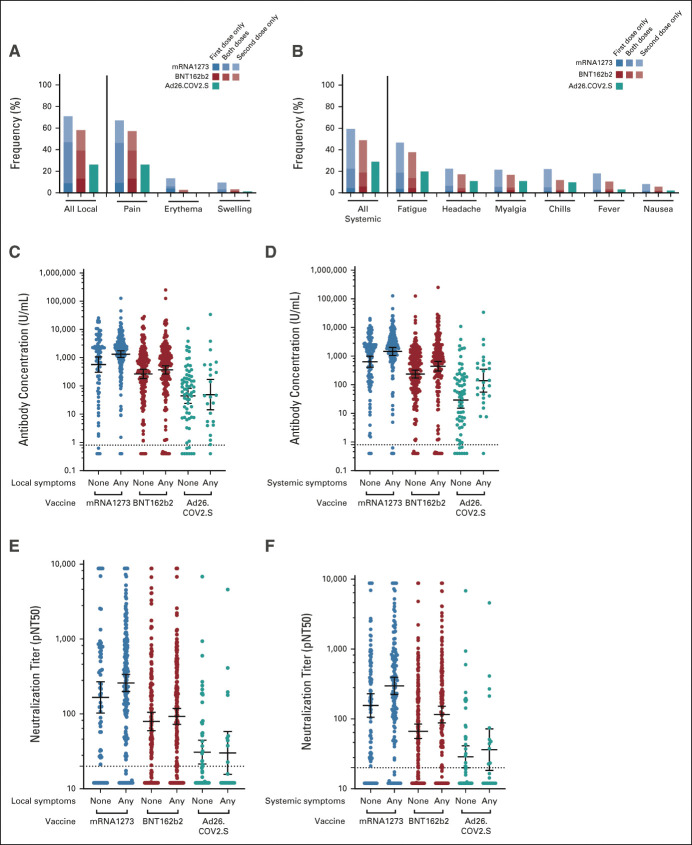
Local (left column) and systemic (right column) reactogenicity of SARS-CoV-2 vaccines and their association with antispike immunoglobulin A/G/M antibody concentrations (middle row) or neutralization titer (bottom row). (A and B) Bars are colored according to vaccine (blue mRNA-1273, red BNT162b2, and teal Ad26.COV2.S) and timing of symptoms: lower solidly shaded box indicates after first dose only, middle moderately shaded box indicates after both doses, and upper lightly shaded box indicates after second dose only. (C-F) Horizontal line denotes the geometric mean concentration or titer and whiskers denote the 95% CI. pNT50, pseudovirus neutralization titer 50.

We assessed local and systemic adverse effects after vaccination. The majority of participants, 71.5% (545 of 762), reported at least one local or systemic symptom after vaccination (Data Supplement). The most frequent local symptom was pain at the site of injection (Data Supplement; Fig 3). The timing of local symptoms was most frequently after both doses of vaccine, or after the second dose only (in mRNA vaccine recipients). The most common systemic symptom was fatigue. Systemic symptoms were most commonly seen after the second dose of vaccine. The frequency of local or systemic symptoms was highest in mRNA-1273 recipients (81%, 233 of 288), followed by BNT162b2 recipients (72%, 274 of 383) and lowest in Ad26.COV2.S recipients (42%, 38 of 91) (*P* < .001; Data Supplement). Most patients reported their symptoms were mild or moderate (89%, 479 of 538). Prior infection was associated with higher systemic, but not local, symptoms (Data Supplement).

### Association Between Reactogenicity and Immunogenicity of SARS-CoV-2 Vaccines

As reactogenic symptoms may be because of immune mechanisms involved in vaccine immunogenicity, we hypothesized that reactogenicity may associate with immunogenicity. Adjusting for vaccine type and prior infection, the presence of systemic symptoms was associated with higher antibody concentration (0.23 log_10_ U/mL higher; CI, 0.08 to 0.39; *P* = .002) and neutralization titers (0.14 log_10_ higher; 0.03 to 0.25; *P* = .016; Fig 3 and the Data Supplement). No association between local symptoms and immune responses was observed.

### Responses to Additional Vaccine Doses

**FIG 4.**
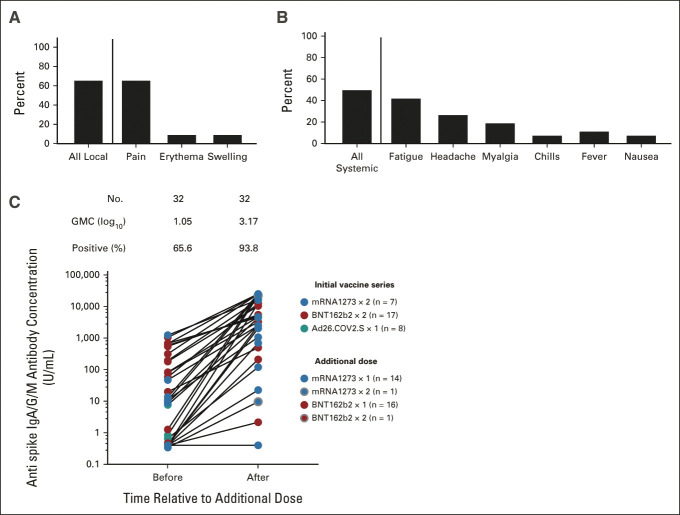
Reactogenicity and immunogenicity of additional doses of SARS-CoV-2 vaccines following completion of the primary series of vaccines (n = 32). (A and B) The frequency of local and systemic symptoms following receipt of an additional vaccine dose among 26 of 32 individuals who completed the questionnaire. (C) The antispike IgA/G/M concentration before and after receipt of a booster dose of vaccination. The color of each dot indicates the initial vaccine series and additional vaccine as indicated in the legend insert. The number of donors, GMC, and proportion positive at a threshold of > 0.8 U/mL are shown above each group. GMC, geometric mean concentration; Ig, immunoglobulin.

The safety and immune response to additional vaccine doses given after the full series of vaccines is not known. Thirty-two participants reported receiving either the mRNA-1273 (n = 15) or BNT162b2 (n = 17) vaccine following completion of the mRNA-1273 (n = 7), BNT162b2 (n = 17), or Ad26.COV2.S (n = 8) series, either on the recommendation of their medical team, or following recent CDC guidance (September 12, 2021). One participant each reported receiving two additional doses of mRNA-1273 or BNT162b2, and are included in the analysis below. We measured responses at a median of 20 (IQR, 16-27) days after receipt of the additional vaccine. The frequency of local side effects was 65% (largely pain at the site of injection) and of systemic side effects was 50% (Fig 4). No patient experienced any severe adverse or allergic reaction; side effects were self-reported as mild or moderate in all cases. Before receipt of the additional dose, the GMC was 1.05 log_10_ U/mL; 11 individuals had negative antibody titers. Antibody concentrations rose in 30 of 32 individuals, but two participants (both patients with chronic lymphocytic leukemia treated with obinutuzumab in the prior year) remained seronegative (Fig 4). The GMC after vaccination was 3.17 log_10_ U/mL, comparable with baseline immunogenicity among healthy (noncancer) recipients of mRNA-1273 or BNT162b2 without prior infection. In an exploratory analyses, the degree of titer increase appeared to vary according to initial vaccine, additional dose, and whether the additional dose was homologous to the initial vaccine (Data Supplement). Neutralization titers rose similarly in the subset of patients with available measures (Data Supplement).

## DISCUSSION

We studied the immunogenicity and reactogenicity of SARS-CoV-2 vaccines in a large, prospective cohort of patients with diverse solid-organ and hematologic malignancies. By assessing both binding antibodies and antibody neutralization, we found that responses to the three vaccines deployed under EAU in the United States are impaired modestly in patients with cancer relative to healthy controls, and vary according to vaccine, age, cancer type, and therapy. The frequency of local or systemic reactions in patients with cancer were similar to rates reported in healthy individuals,^24^ and systemic symptoms were more common in patients with prior infection. Systemic reactogenicity was associated with the magnitude of immune response. Furthermore, additional doses of vaccine among patients with low responses had a favorable side-effect profile and induced immune responses.

There are several noteworthy findings in this study. Consistent with what has been previously observed in healthy controls,^19^ Ad26.COV2.S induced low responses, and few patients had measurable neutralization. We found that patients receiving chemotherapy in the prior year or bone-marrow transplant recipients had lower immune responses, but the magnitude of these effects were small (smaller than that of the vaccine type for example) and most still had neutralization titers predicted to be protective. Interestingly, individuals receiving immune checkpoint blockade tended to have enhanced neutralization. Although widely hypothesized, this latter finding had not been previously reported, to our knowledge. We speculate that this may be because of enhanced CD4+ T-cell help leading to qualitatively improved B-cell activation, affinity maturation, and antibody production. Similarly, although widely hypothesized and assessed in small studies, the association between prior infection and reactogenicity, and between systemic reactogenicity and immunogenicity, has not been robustly assessed until now, yet is important for public health messaging.

These data have several salient clinical implications.^6^ Although the exact correlate of protection has not been determined, animal studies,^23,25^ randomized trials of prophylactic neutralizing antibodies,^26,27^ and correlative studies^6,7^ suggest that neutralizing antibody titer and binding antibody titers are a correlate of protection from infection or severe disease. First, these data reinforce public health messaging that SARS-CoV-2 vaccines are safe—even in an oncology patient population. Side effects to vaccines are mild or moderate and correlate with enhanced responses. Most individuals with cancer achieve responses that are likely to be sufficient to protect against severe disease.^6^ Second, given differences in immune responses based upon vaccine type (eg, 69% of patients who received Ad26.COV2.S had undetectable neutralization), our data suggest that where options exists, mRNA vaccines be prioritized for patients with cancer. Moreover, patients who received the Ad26.COV2.S vaccine should be considered for additional vaccine doses. The higher immunogenicity of mRNA1273 compared with BNT162b2 may plausibly be attributable to the higher administered dose of the former. Third, chemotherapy exposure appeared to have a long-lasting, albeit modest, impact on immunogenicity; larger studies may be required to understand whether holding chemotherapy around vaccination affects immunogenicity. Fourth, corticosteroids appeared to blunt binding antibody titers but did not significantly affect neutralization titers. Finally, additional booster vaccine doses appear well tolerated in patients with cancer, as has been observed in solid-organ transplant recipients,^28,29^ and are capable of inducing immune responses comparable with those achieved by healthy individuals after the primary vaccine series (in patients not on B-cell–depleting agents). Collectively, these data suggest that antibody testing may help identify individuals who may be candidates for additional doses of vaccine.

This study has some important limitations. (1) Measures of immune response serve as a surrogate measure of protection, and the ultimate outcome of interest is whether patients develop breakthrough SARS-CoV-2 infection, and the severity thereof. (2) We assessed neutralization of the original Wuhan strain of SARS-CoV-2 but the beta, gamma, and delta viral variants show several-fold lower neutralization in other studies.^18,30^ (3) Although prospectively assessed, we cannot exclude unmeasured confounding explaining differences in immunogenicity between patient groups. (4) We report baseline vaccine response here; assessment of responses at additional timepoints will allow for more robust estimation of antibody decay rates. (5) We enrolled few patients receiving B-cell–depleting agents, in whom other studies have demonstrated low responses.^13-15^ (6) We did not assess T-cell responses whose role remains unclear in preventing infection. (7) The number of patients who recieved additional doses was small, thus limiting study of the correlates of response. Further studies to address these limitations are needed.

In summary, SARS-CoV-2 vaccines are well tolerated in patients with cancer, and most recipients achieve responses that are likely to be associated with protection. We define how receipt of specific vaccines or some therapies may impair responses. Studies on the effectiveness of vaccines in preventing breakthrough infection and the potential benefit of additional vaccine doses are required.
